# A transcriptional view on somatic embryogenesis

**DOI:** 10.1002/reg2.91

**Published:** 2017-12-05

**Authors:** Anneke Horstman, Marian Bemer, Kim Boutilier

**Affiliations:** ^1^ Bioscience Wageningen University and Research Wageningen The Netherlands; ^2^ Laboratory of Molecular Biology Wageningen University and Research Wageningen The Netherlands

**Keywords:** chromatin modifications, regeneration, somatic embryogenesis, totipotency, transcription factors

## Abstract

Somatic embryogenesis is a form of induced plant cell totipotency where embryos develop from somatic or vegetative cells in the absence of fertilization. Somatic embryogenesis can be induced in vitro by exposing explants to stress or growth regulator treatments. Molecular genetics studies have also shown that ectopic expression of specific embryo‐ and meristem‐expressed transcription factors or loss of certain chromatin‐modifying proteins induces spontaneous somatic embryogenesis. We begin this review with a general description of the major developmental events that define plant somatic embryogenesis and then focus on the transcriptional regulation of this process in the model plant *Arabidopsis thaliana* (arabidopsis). We describe the different somatic embryogenesis systems developed for arabidopsis and discuss the roles of transcription factors and chromatin modifications in this process. We describe how these somatic embryogenesis factors are interconnected and how their pathways converge at the level of hormones. Furthermore, the similarities between the developmental pathways in hormone‐ and transcription‐factor‐induced tissue culture systems are reviewed in the light of our recent findings on the somatic embryo‐inducing transcription factor BABY BOOM.

## 1 INTRODUCTION

Plants are developmentally plastic organisms. Not only do they continually differentiate new organs from the stem cell niche throughout their lifespan, but they also regenerate new cells and organs after wounding or during in vitro culture (pluripotency) (Ikeuchi, Ogawa, Iwase, & Sugimoto, 2016, and references therein). Plant cells also show an extraordinary capacity for totipotent growth, the ability to produce a new organism through embryogenesis. During sexual reproduction, a diploid zygote is formed upon fusion of two haploid gametes, an egg cell and a sperm cell, and goes on to form the embryo and eventually a new plant. In flowering plants, the embryo develops together with the endosperm, and both are surrounded by the maternally derived seed coat. Together these tissues constitute a seed. During germination, the embryo breaks out of the seed coat and develops further to produce the different organs that make up the plant body. Thus the single‐celled zygote has the capacity to form a whole plant and is therefore totipotent. A number of plants produce embryos in the absence of egg cell fertilization as part of their natural reproductive cycle. In apomictic plants, embryos develop spontaneously from the sporophytic tissues of the seed coat precursor or from an unreduced gametophytic cell. Adventitious plantlets also form on the leaf margins of some plants, e.g., *Kalanchoë* spp., also known as ‘mother of thousands.’ In *K. daigremontiana* these plantlets initiate through adventitious embryogenesis and then complete their development through organogenesis (Garces et al., 2007). The capacity for totipotent growth reaches its maximum potential during in vitro tissue culture, where an even wider range of explants can be induced to undergo embryogenesis, including haploid cells of the male and female gametophyte (gametophytic embryogenesis) (Soriano, Li, & Boutilier, 2013) and vegetative cells of the sporophyte (somatic embryogenesis, SE) (Elhiti, Stasolla, & Wang, 2013).

Plant totipotency research has its history in the cell theory of Schleiden (Schleiden, 1838) and Schwann (Schwann, 1839), which states that organisms comprise individual cells that have the capacity to grow and divide independently. Building on this theory, Haberlandt laid the foundation for in vitro plant totipotency research by predicting that artificial embryos could be generated from cultured cells (Haberlandt, 1902). In vitro SE was first described experimentally almost 60 years later, by Waris, working on *Oenanthe aquatic* (water dropwort) (Waris, 1957; as reviewed in Krikorian & Simola, 1999; Vasil, 2008) and shortly thereafter by Steward et al. and Reinert, both working on *Daucus carota* (carrot) (Reinert, 1958; Steward, Mapes, & Mears, 1958). Evidence for the totipotency of cultured gametophytes soon followed, when Guha and Maheshwari produced haploid embryos from the pollen grains of *Datura innoxia* Mill. anthers (Guha & Maheshwari, 1964, 1967).

At that time, scientists working in the field had already recognized the potential of in vitro embryogenesis as a plant propagation tool. In vitro embryogenesis is now a standard biotechnology tool, with applications in both industrial and academic laboratories. Somatic embryos retain the genotype and ploidy of the donor explant and are used to clonally propagate plants for different applications, including scaling‐up of breeding material for testing, and for shortening the breeding cycle of highly heterozygous plants and plants with long life cycles. It is also used to regenerate transgenic plants after transformation. SE is often the preferred clonal propagation tool for plant breeding, as plantlets can be obtained in a single step rather than through the sequential regeneration of shoots and roots that is required during de novo organogenesis. The higher throughput of this technique and the potential for storage of cultures and embryos also contribute to its utility over de novo organogenesis and rooted cuttings. Such advantages have led to significant increases in production efficiency and uniformity, and in the quality of crop germplasm, especially in the forestry sector (Lelu‐Walter et al., 2013; Park, 2002). Despite the many advantages, the use of SE for clonal propagation can be limited by the production of ‘off‐types,’ resulting from somatic mutations or stable chromatin modifications (Miguel & Marum, 2011).

Haploid embryos are also used as a plant propagation tool. Spontaneous or chemical doubling of haploid embryos generates fertile diploid plants that are homozygous at all loci, so called doubled‐haploid plants. The ability to obtain a fully homozygous plant in a single generation is routinely exploited in plant breeding programs, where doubled‐haploid plants are used to produce homozygous parent lines for F_1_ hybrid seed production, to accelerate backcross conversion and to develop genetic marker maps (reviewed in Dwivedi et al., 2015; Forster, Heberle‐Bors, Kasha, & Touraev, 2007; Germanà, 2011).

One of the major bottlenecks facing widespread application of in vitro embryogenesis as a plant propagation tool is the low responsiveness of many species and genotypes. This recalcitrance affects not only embryo induction, but also the subsequent steps in the regeneration process, including chromosome doubling (in the case of haploid embryos), histogenesis (differentiation), and conversion (germination) from embryo to plantlet.

The in vitro embryogenesis field has developed through the years to include studies aimed at obtaining and improving embryogenesis, as well as studies aimed at understanding how in vitro embryogenesis is initiated and maintained. What has become clear is that, although gametophytic and somatic embryos differ in origin, they share many commonalities, including the treatments used to induce embryogenesis and the developmental changes that lead to the production of embryos (Hand, de Vries, & Koltunow, 2016; Verdeil, Alemanno, Niemenak, & Tranbarger, 2007), suggesting that the two processes represent different faces of the same coin. How these different types of totipotent growth are induced at the molecular level, where these developmental pathways converge, and their relation to natural totipotency and other forms of in vitro regeneration (pluripotency) are major questions in plant biology. In this review, we make use of the large body of information available for the model plant *Arabidopsis thaliana* (L.) Heyhn. (arabidopsis) to develop a framework for understanding plant SE. We begin with a general explanation of the different developmental concepts and events that define SE, describe the different SE systems developed for arabidopsis, and then discuss the roles of transcription factors (TFs) and chromatin‐modifying proteins in this process. Where relevant, we also include observations on other regeneration systems and model plants to augment this overview.

## 2 DEFINITIONS AND CONCEPTS

SE can be induced in a wide range of explants, most commonly by treating them with plant growth regulators, usually the synthetic auxin 2,4‐dichlorophenoxyacetic acid (2,4‐D) and/or abiotic stress treatments. The mechanism underlying auxin‐ and stress‐induced SE is not known, but both treatments induce biosynthesis of endogenous auxins, which is thought to be an important early step in the switch to totipotent growth (Feher, 2015).

Somatic embryos, as with all in vitro and zygotic embryos, are bipolar structures with an apical pole (the future shoot) and a basal pole (the future root), each with its own meristem, and an independent provascular system. This bipolarity distinguishes somatic embryos from ectopic or adventitious organs, such as shoots and roots, which are unipolar structures with a (lignified) vascular connection to the underlying explant. Somatic embryos also accumulate species‐specific storage products that are not found at other stages of plant development, although the extent to which they do so is often determined by the ability of the culture conditions to mimic the storage product accumulation phase of seed development. The absence of trichomes, which can be found on the first leaves of some plants, is often used as a morphological marker for somatic embryo formation, although trichome formation can be misleading as it can be delayed initially.

### 2.1 Direct and indirect somatic embryogenesis

**Figure 1:**
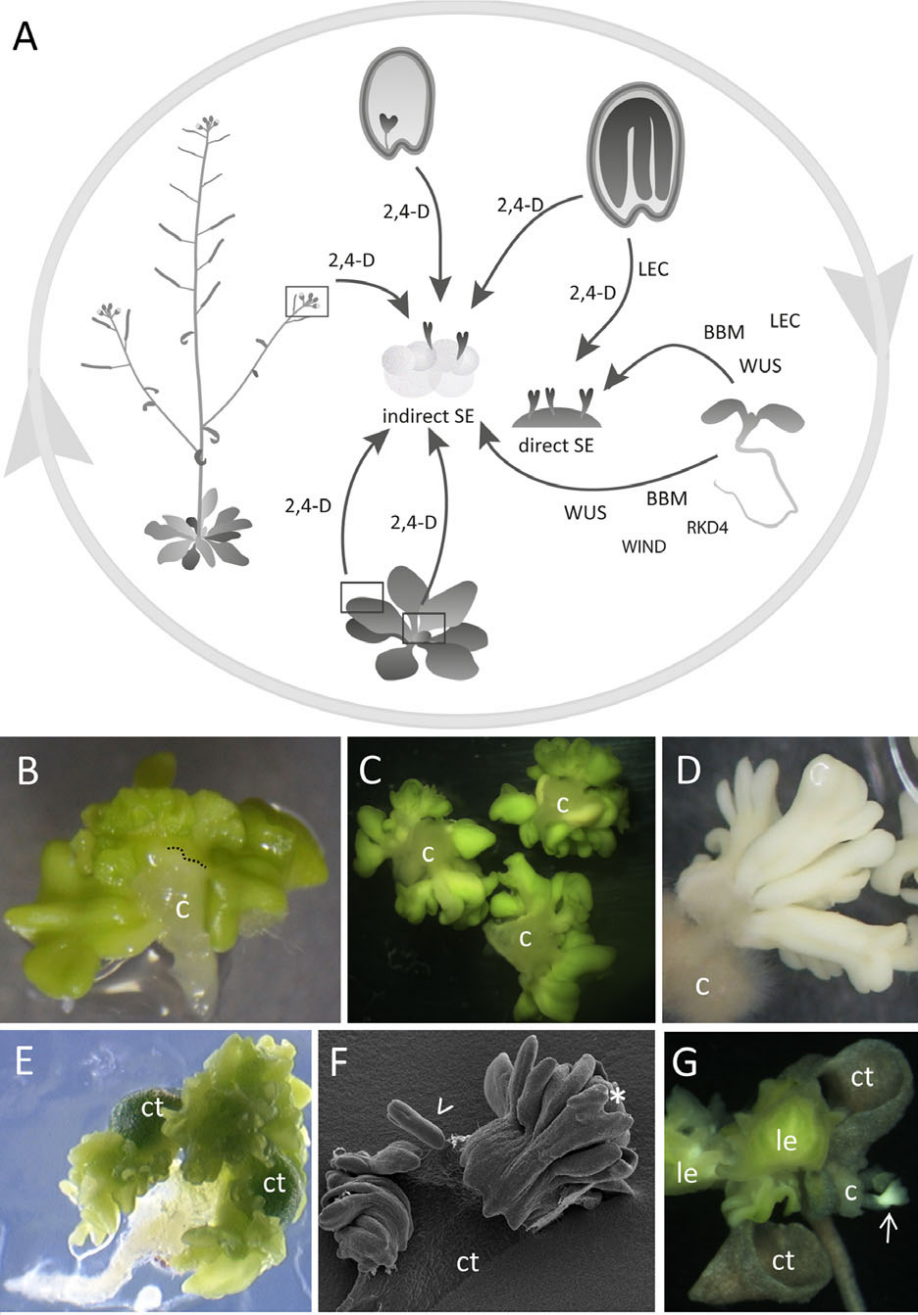
Somatic embryogenesis in arabidopsis. (A) Schematic overview of somatic embryogenesis (SE) systems in arabidopsis. SE can be induced from a range of tissues throughout the arabidopsis life cycle, and proceeds either directly or indirectly via a callus phase. SE can be induced using the synthetic auxin 2,4‐D or by overexpression of specific transcription factors, including BABY BOOM (BBM), LEAFY COTYLEDON (LEC), WUSCHEL (WUS), RWP‐RK DOMAIN‐CONTAINING 4 (RKD4) and WOUND INDUCED DEDIFFERENTIATION 1 (WIND1). (B)−(F) SE from different arabidopsis explants. (B) Direct SE from immature zygotic embryos treated with 2,4‐D on solid medium. Embryos develop from the edge of the original explant (dotted line), while the underlying tissue forms callus. (C) Indirect SE from immature zygotic embryos treated with 2,4‐D in liquid medium. (D) Secondary SE. Callus from primary somatic embryos cultured in liquid medium produces secondary somatic embryos after removal of 2,4‐D (Su et al., 2009). (E) *35S:BBM* induces direct SE on the cotyledons and shoot meristem of germinated seedlings. (F) Scanning electron micrograph showing somatic embryo development on a *35S:BBM* cotyledon. The embryos develop directly from the explant (>), are bipolar and undergo direct secondary embryogenesis (*). (G) Indirect SE on a *35S:BBM‐GR* seedling. Embryos (arrow) develop from callus produced on the cotyledons. c, callus; ct, cotyledon; le, leaf

The developmental steps that take place after a cultured explant is induced to undergo SE have been well described at the histological level. Two developmental routes can be followed, termed indirect and direct embryogenesis (Fig. 1), although in practice it can be difficult to distinguish between the two, and both often occur on the same explant. Indirect SE is the most common pathway, and starts with the formation of a callus, a seemingly unorganized mass of initially vacuolated cells that show different degrees of compactness (Ikeuchi, Sugimoto, & Iwase, 2013). Due to its initially amorphous structure, callus was (and often still is) referred to as “undifferentiated” or “dedifferentiated,” but these terms are rather ambiguous in the absence of more precise molecular information. A number of pioneering studies in arabidopsis show that organogenic callus (callus used for adventitious shoot production) has a lateral root identity, and like lateral roots is derived from stem‐cell‐like pericycle cells (Atta et al., 2009; Che, Lall, & Howell, 2007; Sugimoto, Jiao, & Meyerowitz, 2010). This suggests that one of the earliest steps in de novo shoot organogenesis involves cell redifferentiation to a distinct cell type, rather than to an “undifferentiated/dedifferentiated” state. Embryogenic callus develops from both pericycle and non‐pericycle cells (Guzzo et al., 1995; Raghavan, 2004), but it is not known whether this callus has lateral root identity. Embryogenic callus formation is followed by the development of proembryogenic masses (PEMs) on the surface or within the callus mass, from which single cells or cell clusters develop into embryos (Halperin, 1966; Toonen et al., 1994). Callus and PEMS are usually observed after treatment with auxins, especially 2,4‐D, and while auxin promotes callus and PEM initiation and proliferation, it usually needs to be removed to promote histogenesis (apical−basal and bilateral patterning) and elongation of the embryo.

The second pathway, direct embryogenesis, is less well defined, as it is characterized by the absence of a callus phase. In this system, the explant shows less prolific and more regular compact cell divisions. Single or multiple cells in single or multiple cell layers divide and bulge to develop into morphologically recognizable embryos without further treatment (Williams & Maheswaran, 1986). Somatic embryos from the direct and indirect pathways are morphologically similar, but genome‐level changes (somaclonal variation) often occur in embryos derived from indirect SE due to the longer tissue culture period (Miguel & Marum, 2011). Somatic embryos can also be used to induce a new round of SE, termed secondary or cyclic embryogenesis (Raemakers, Jacobsen, & Visser, 1995). Secondary embryos can be induced directly from the primary embryos or indirectly after embryogenic callus formation.

The ability of an explant to undergo direct or indirect embryogenesis was historically thought to be determined by the age of the explant: the further the explant is from the zygotic embryo stage, the more reprogramming (callus formation) is required to convert the explant into a somatic embryo (Merckle, Parrott, & Flinn, 1995). Although it is often more difficult to obtain somatic embryos from developmentally older tissues and organs, when somatic embryos develop, they can develop by either the direct route or the indirect route regardless of the age of the tissue (Dubois, Guedira, Dubois, & Vasseur, 1990; Gaj, 2004; Guzzo et al., 1995). It seems that the developmental context of a cell or tissue in combination with the culture environment is more important in defining whether embryogenesis is direct or indirect than its developmental distance from the embryonic state.

### 2.2 Characteristics of embryogenic cells

How (and when) are embryogenic cells formed in tissue culture? Regardless of their origin, single or multiple cells in the explant can be converted into cells with embryo identity, but whether this involves direct transdifferentiation to an embryogenic state or redifferentiation to one or more intermediary identities is not known (Radoeva & Weijers, 2014). Historically, PEMs and embryogenic cells have been defined using histological techniques. They can be distinguished from the surrounding cells/callus by their relatively high nucleus to cytoplasm ratio, a nucleus with a single large nucleolus and relatively low heterochromatin levels, the presence of fragmented vacuoles, and by their callose‐containing cell walls (Verdeil et al., 2007). Although we have a good idea about the types of cells that form somatic embryos in vitro (Filonova, Bozhkov, & Arnold, 2000; Toonen et al., 1994) and the cellular characteristics of these cells (Emons, 1994; Verdeil et al., 2007; Yeung, 1995), the process remains largely undescribed at the molecular level. High throughput expression analyses have been used to identify characteristics of embryogenic explants (Salvo, Hirsch, Buell, Kaeppler, & Kaeppler, 2014; Trontin, Klimaszewska, Morel, Hargreaves, & Lelu‐Walter, 2016; Wickramasuriya & Dunwell, 2015; Yang et al., 2012), but most studies use whole explants, which contain a complex mixture of tissues and cell types, making it difficult to specifically assign molecular identities to embryogenic cells. In situ gene expression analyses would help to resolve the sometimes contradictory relationship between expression of developmental marker genes and cell fate in different culture systems (Miguel & Marum, 2011) and provide a more exact description of when and how embryogenic cells are formed in culture (Li et al., 2014; Soriano et al., 2014).

## 3 ARABIDOPSIS AS A MODEL SYSTEM

By far the majority of research on SE is focused on protocol development. However, a number of model systems are also being used to understand the mechanism driving somatic embryo induction, including carrot, Norway spruce, alfalfa, cotton, and arabidopsis. Arabidopsis has evolved into one of the best systems to study SE due to the availability of efficient protocols for direct and indirect SE from different explants, and the wealth of cell biology and functional genomics tools. The development of arabidopsis as a model system was also fueled by the discovery that ectopic expression of meristem and embryo regulatory proteins, as well as loss‐of‐function mutants in chromatin‐modifying proteins, promote somatic embryo development in seedlings, and that many of these proteins also have a role in 2,4‐D‐induced SE (Feher, 2015). Below we describe the different somatic embryo culture systems that have been developed for arabidopsis and then focus specifically on the role of TFs and chromatin‐modifying proteins in regulating SE.

### 3.1 Tissue culture systems

Several in vitro somatic embryo systems have been developed for arabidopsis that encompass a wide range of explants, including immature zygotic embryos (Gaj, 2001; Ikeda‐Iwai, Satoh, & Kamada, 2002; Mordhorst et al., 1998; Sangwan, Bourgeois, Dubois, & Sangwannorreel, 1992), mature zygotic embryos (dry seeds) (Kobayashi, Nagayama, Higashi, & Kobayashi, 2010), leaf protoplasts (Luo & Koop, 1997; O'Neill & Mathias, 1993), shoot apices and flower buds (Ikeda‐Iwai, Umehara, Satoh, & Kamada, 2003) (Fig. 1). In general, the synthetic auxin 2,4‐D is used to induce SE, although a short heavy metal, salt or osmotic stress treatment, alone or followed by culture in 2,4‐D, can be used to induce SE from seedling shoot apices (Ikeda‐Iwai et al., 2003). Primary somatic embryos induced by 2,4‐D treatment can also be used as explants to produce secondary somatic embryos via embryogenic callus (Ikeda‐Iwai et al., 2002; Pillon, Terzi, Baldan, Mariani, & LoSchiavo, 1996; Su et al., 2009).

The most extensively used and studied arabidopsis SE system uses 2,4‐D‐treated immature zygotic embryos at the bent cotyledon stage of development as the explant (Fig. 1). Depending on the culture conditions, somatic embryos develop directly from the explant, or indirectly from callus on the adaxial side of the cotyledon, and often develop later from callus formed on the abaxial side of the cotyledons (Kurczynska, Gaj, Ujczak, & Mazur, 2007; Raghavan, 2004). At least two parameters influence whether direct or indirect SE will take place: the age of the explant and the use of solid or liquid medium (Gaj, 2004, 2011). Early globular to bent cotyledon‐stage embryos (>400–500 μm) undergo predominantly indirect SE, while older bent cotyledon‐stage embryos (>500 μm) undergo predominantly direct SE (Gaj, 2001). However, immature zygotic embryo explants that give >80% direct SE on solid medium will undergo indirect SE when grown in the same liquid medium (Gaj, 2011; Mordhorst et al., 1998). In the direct system, differentiated somatic embryos develop without further treatment, but in the indirect system removal of 2,4‐D is required to promote (better) embryo differentiation and elongation.

### 3.2 Totipotency and pluripotency go hand in hand

The formation of cytoplasmically rich clusters and early somatic embryos in explants undergoing direct SE coincides with the expression of a number of zygotic embryo markers (Wickramasuriya & Dunwell, 2015). Two embryo marker genes, *LEAFY COTYLEDON2* (Kurczynska et al., 2007) and *BABY BOOM* (*BBM*) (Kulinska‐Lukaszek, Tobojka, Adamiok, & Kurczynska, 2012) (see below) are expressed in proliferating epidermal/subepidermal cells from which somatic embryos eventually develop, suggesting that these cell clusters have acquired embryo identity. The changes in tissue histology and the slow appearance of embryo marker gene expression (around 5−6 days after the start of culture) suggest that the transition to embryo identity is achieved gradually through one or more intermediate developmental states rather than through direct transdifferentiation of existing cell types. Similar studies on the timing of embryo fate establishment have not been performed in indirect SE systems.

The developmental context in which arabidopsis SE takes place is complex, as both somatic embryos and adventitious shoots can develop side by side in the same explant. Bassuner, Lam, Lukowitz, and Yeung (2007) examined the histology of adventitious shoot and root formation during indirect SE from immature zygotic embryo explants. Single or fused shoots were connected to the explant by a broad tissue base, whereas somatic embryos developed from isolated cell clusters with only a weak connection to the explant. The authors hypothesized that the lack of connection between the somatic embryo and underlying explant might prevent auxin flow back into the explant, allowing establishment of a root meristem and a bipolar embryo, while the broader connection observed in shoots would allow auxin canalization/transport and formation of a continuous vascular connection with the explant. Earlier, Raghavan (2005) examined the relationship between the duration of 2,4‐D exposure and cell fate in the indirect SE system. Culture of immature zygotic embryos on 2,4‐D for different periods of time before transfer to 2,4‐D‐free medium induced a continuum of morphogenetic changes from callus. A short auxin exposure induced adventitious shoot formation, a medium auxin exposure induced somatic embryo formation and somatic embryos in which the cotyledons were converted to leaves, and a long auxin exposure induced only somatic embryo formation. Although these two studies are somewhat contradictory (adventitious shoots were observed after extended auxin treatment in the study by Bassuner et al.), they suggest that adventitious shoot and somatic embryo formation represent a developmental continuum, and that a threshold auxin concentration and/or threshold of auxin signaling are important for inducing and maintaining embryo identity in vitro.

A similar phenomenon, where totipotent and pluripotent growth occur side by side, is also observed during TF‐induced SE, and molecular analysis of these pathways has shed some light on how the processes are controlled.

## 4 A NETWORK OF ARABIDOPSIS TRANSCRIPTION FACTORS CONTROLS SOMATIC EMBRYO INDUCTION

A number of TFs have been identified that induce spontaneous SE when ectopically expressed in arabidopsis seedlings, i.e., without the stress or growth regulator treatments that are required in wild‐type arabidopsis. Recent studies on the genetic and molecular interactions between these TFs and their role in 2,4‐D‐mediated SE have revealed an interacting network that acts on hormone pathways. Below, we describe these TFs and their molecular interactions.

### 4.1 Ectopic expression of embryo and meristem identity genes can induce somatic embryogenesis

The LEAFY COTYLEDON (LEC) proteins LEC1 and LEC2 were the first TFs shown to induce SE when ectopically expressed in seedlings (Lotan et al., 1998; Stone et al., 2001). LEC1, which encodes subunit B9 of a nuclear factor Y protein (NF‐YB9), and the B3 domain protein LEC2 are part of a larger network of “LAFL” proteins (for LEC1/LEC1‐LIKE [L1L], ABSCISIC ACID [ABA] INSENSITIVE 3 [ABI3], FUSCA3 [FUS3] and LEC2) that regulate embryo identity and maturation (Jia, McCarty, & Suzuki, 2013). Loss‐of‐function mutations in *LAFL* genes result in defects in cotyledon development, storage macromolecule accumulation, and desiccation tolerance in zygotic embryos (Keith, Kraml, Dengler, & McCourt, 1994; Meinke, Franzmann, Nickle, & Yeung, 1994; Parcy, Valon, Kohara, Misera, & Giraudat, 1997; Stone et al., 2001; West et al., 1994). In contrast, ectopic expression of *LEC1* and *LEC2* induces somatic embryo formation on the cotyledons and leaves of arabidopsis seedlings (Lotan et al., 1998; Stone et al., 2001). Later it was found that L1L/NUCLEAR FACTOR Y subunit B6 (NF‐YB6) and three other NF‐Y subunits, A1, 5 and 9, with roles in embryo development, drought resistance, and ABA perception (Kwong et al., 2003; Li et al., 2008; Warpeha et al., 2007), also induce spontaneous SE in seedlings when overexpressed (Mu, Tan, Hong, Liang, & Zuo, 2013). The remaining two *LAFL* genes, *FUS3* and *ABI3*, do not induce SE when overexpressed, but do confer cotyledon identity to leaves (Gazzarrini, Tsuchiya, Lumba, Okamoto, & McCourt, 2004; Parcy et al., 1994).

Another embryo‐expressed TF that can induce SE is RWP‐RK DOMAIN‐CONTAINING 4 (RKD4)/GROUNDED (GRD) (Waki, Hiki, Watanabe, Hashimoto, & Nakajima, 2011). *RKD4* is expressed throughout early embryos and in suspensors. While mutation of *RKD4* leads to short suspensors and embryo arrest, induced overexpression of *RKD4* in seedlings causes overproliferation of root cells, from which somatic embryos developed. In line with its unique role during early embryogenesis—other RKDs only affect embryo sac development—RKD4 is the only RKD factor that induces SE (Koszegi et al., 2011; Tedeschi, Rizzo, Rutten, Altschmied, & Baumlein, 2017; Waki et al., 2011).

BBM is a member of the *AINTEGUMENTA‐LIKE* (*AIL*) clade of AP2/ERF TFs that was initially identified as a marker for the induction of haploid embryo development from *Brassica napus* immature pollen grains (Boutilier et al., 2002). Ectopic expression of *BBM* is sufficient to induce SE on the leaves and cotyledons of arabidopsis seedlings without exogenous hormone application (Boutilier et al., 2002). *BBM* overexpression also induces other types of regeneration, including callus and adventitious shoot and root formation. This property has been exploited to improve transformation in crop and model plants (Deng, Luo, Li, & Yang, 2009; Florez, Erwin, Maximova, Guiltinan, & Curtis, 2015; Heidmann, de Lange, Lambalk, Angenent, & Boutilier, 2011; Lutz, Azhagiri, & Maliga, 2011).

*BBM* belongs to a gene clade that also includes *AINTEGUMENTA* (*ANT*) and six other *AIL*/*PLETHORA* (*PLT*) genes (Horstman, Willemsen, Boutilier, & Heidstra, 2014). Arabidopsis *BBM* and the other arabidopsis *AIL*/*PLT* genes are expressed in the embryo and the root and/or shoot meristems, where they act redundantly to maintain embryo growth and to define and maintain the stem cell niches (Aida et al., 2004; Galinha et al., 2007; Mudunkothge & Krizek, 2012). Overexpression of *AIL5* also triggers somatic embryo and adventitious organ formation (Tsuwamoto, Yokoi, & Takahata, 2010). Recently, it became clear that overexpression of all AIL proteins, except the phylogenetically distinct AIL1 and ANT, induces SE (Horstman et al., 2017). This shows that the embryo‐inducing capacity of AIL proteins is not limited to embryo‐expressed AILs, and suggests that AIL proteins can regulate similar target genes.

Overexpression of another member of the AP2/ERF TF family, *WOUND INDUCED DEDIFFERENTIATION 1* (*WIND1*) or *RAP2.4*, also induces SE (Ikeuchi et al., 2013). *WIND1* and its close homologs *WIND2−4* are induced by wounding and stimulate callus proliferation after tissue damage (Iwase et al., 2011). Ectopic *WIND1* expression is sufficient to promote callus formation from shoots, hypocotyls, and roots (Iwase et al., 2011), which can then give rise to shoots, roots, or somatic embryos (Ikeuchi et al., 2013).

*WUSCHEL* (*WUS*) is a homeodomain TF that is expressed in flower and shoot meristems, where it induces stem cell fate in a non‐cell‐autonomous manner (Laux, Mayer, Berger, & Jurgens, 1996; Mayer et al., 1998). Overexpression of *WUS* in arabidopsis is sufficient to induce organogenesis and SE in the shoot and root tip (Chatfield et al., 2013; Gallois, Nora, Mizukami, & Sablowski, 2004). *WUS* was also identified as *PLANT GROWTH ACTIVATOR 6* (*PGA6*) in an activation tagging screen for genes that induce somatic embryo formation from root callus (Zuo, Niu, Frugis, & Chua, 2002).

The above studies show that SE can be induced by ectopic expression of TFs from several different classes, with different roles during plant development. Some of these TFs have roles in early embryo development or in maintaining embryo identity, but non‐embryo‐expressed stem cell regulators can also induce SE. Below we compare the regeneration pathways that are induced by overexpression of these different TFs.

### 4.2 Regeneration pathways

Hormone‐ or stress‐induced SE from cultured explants follows two routes depending on the stage of the explant and the tissue culture conditions. Somatic embryos develop either directly from the explant or indirectly from callus, but it is not always clear why somatic embryos form via one route or the other. These two routes are also observed in TF‐induced SE and studies on these pathways have shed light on this phenomenon.

BBM can induce both direct and indirect SE, depending on the developmental stage of the tissue (Boutilier et al., 2002; Passarinho et al., 2008). Using chemical activation of BBM at different time points, we showed that BBM induces direct SE when activated in a narrow time window before seed germination and indirect SE from callus when activated shortly after germination (Horstman et al., 2017). Direct SE proceeds quickly and the initial steps involve direct activation of *LAFL* gene expression (see below). Indirect SE proceeds more slowly and is not initially associated with BBM‐mediated *LAFL* expression. *LEC1* expression was only detected once embryos started to appear from the callus. At the seed‐to‐seedling transition, *LAFL* genes are repressed by chromatin‐modifying proteins (see below). The inability of BBM to directly activate *LAFL* genes after germination suggests that the chromatin state of these loci might be one factor that determines whether embryos are formed via direct or indirect SE. Constitutive overexpression of *LEC1*, *L1L* or *LEC2* in arabidopsis appears to induce direct SE, as a callus phase has not been described (Lotan et al., 1998; Mu et al., 2013; Stone et al., 2001). Chemical activation of LEC2 in 7‐day‐old seedlings induced embryo characteristics in leaves, but did not induce SE (Feeney, Frigerio, Cui, & Menassa, 2013), suggesting that seedlings at this developmental stage have already lost their competence for SE. Chemical activation of LEC1 within 48 h after imbibition induced small seedlings that consist of smooth, swollen, somatic embryo‐like tissue, while LEC1 activation just after germination induced embryo‐like tissue only from the primary root meristem, and LEC1 activation 4 d after imbibition had no effect at all (Junker et al., 2012). However, embryogenic leaf structures developed at the shoot apex when LEC1 was activated in 10‐day‐old seedlings in the presence of ABA (Junker et al., 2012). ABA levels decrease as embryos convert to seedlings, suggesting that endogenous ABA levels are sufficient for LEC1‐induced SE during seed germination, but not at later time points. BBM does not require ABA to induce SE after germination and does not appear to target genes in the ABA pathway (Horstman et al., 2017), suggesting that BBM regulates target genes in a different pathway to switch cells of older seedlings to a competent state.

Organogenic callus from roots and above‐ground tissues proceeds via a lateral root pathway, starting from pericycle‐like cells around the vasculature (Atta et al., 2009; Che et al., 2007; Sugimoto et al., 2010). It is not known whether the callus that precedes TF‐induced indirect SE originates from pericycle cells or whether this callus has lateral root identity. The embryogenic callus formed during BBM‐induced indirect SE is derived from the outer layers of the cotyledon in the regions above the vasculature, rather than from pericycle‐like cells (Horstman et al., 2017). Both the ground and vascular tissue also form callus in the same explant, but somatic embryos do not seem to develop from this callus. BBM regulates root meristem identity; therefore it would be interesting to determine whether BBM‐induced callus develops via a lateral root pathway.

Not only the timing of expression, but also the protein dose seems to define the developmental response of a tissue to BBM expression. By modulating the amount of nuclear‐localized BBM we showed that a relatively low BBM dose inhibits cell differentiation, a relatively medium dose induces root and shoot organogenesis and a relatively high dose induces SE (Horstman et al., 2017). Conceivably, this dose‐dependent regeneration could be achieved by transcriptional regulation of specific BBM target genes at different BBM doses or by different expression levels of the same target genes.

The effect of WUS on regeneration capacity has been investigated in several studies. Ectopic *WUS* expression appears to have different outcomes depending on the tissue type and auxin concentration. In one study, indirect SE was observed in the presence of exogenous auxin, and direct SE and organogenesis without it (Zuo et al., 2002). In another study, direct SE was observed in the presence of exogenous auxin, while only ectopic shoots were observed without (Gallois et al., 2004). The apparent discrepancy between these two studies might be explained by a dose effect of WUS, similar to that observed for BBM, as in the absence of auxin independent *WUS* overexpression lines exhibit different frequencies of direct and/or indirect SE in roots (M. Bemer and U. Grossniklaus, unpublished data).

The co‐occurrence of callus, adventitious organs and somatic embryos in seedlings that overexpress regeneration‐promoting TFs (*LEC2*, Stone et al., 2001; *WIND1*, Ikeuchi et al., 2013; *BBM*, Boutilier et al., 2002, Horstman et al., 2017; *WUS*, Zuo et al., 2002, Gallois et al., 2004) parallels observations in 2,4‐D SE cultures and lends further support to the idea that SE and organogenesis are very closely related processes that represent different outputs of a developmental continuum.

### 4.3 Transcriptional networks

A number of chromatin immunoprecipitation and gene expression studies have been performed in the past decade to identify the pathways that control TF‐induced somatic embryo formation in arabidopsis. These studies have shown that SE‐inducing TFs regulate common pathways, in particular the auxin pathway, and that there is transcriptional cross‐talk between these different TFs.

Microarray analysis of LEC2 overexpression seedlings identified several target genes (Braybrook et al., 2006; Stone et al., 2008) among which were auxin pathway genes and the *AGAMOUS‐LIKE 15* (*AGL15*) TF gene. LEC2 activates expression of *TRYPTOPHAN AMINOTRANSFERASE OF ARABIDOPSIS 1* (*TAA1*) and the *YUCCA2* and *4* (*YUC*) genes, which encode key enzymes in the auxin biosynthesis pathway (Zhao, 2014). *LEC2* overexpression can compensate for a suboptimal amount of 2,4‐D or for auxins that are less efficient in somatic embryo induction, such as IAA or NAA (Wojcikowska et al., 2013). Conversely, ectopic overexpression of *LEC2* in combination with a normal 2,4‐D concentration is detrimental for somatic embryo production, with callus being produced instead of somatic embryos (Ledwon & Gaj, 2009; Wojcikowska et al., 2013). LEC1 also binds to and activates expression of *YUC10* in seedlings, but only when they are treated with ABA (see above) (Junker et al., 2012). The importance of endogenous auxin production for 2,4‐D‐induced SE was demonstrated using *yuc* knockout mutants, which show a reduced response in 2,4‐D‐treated secondary somatic embryo cultures (Bai, Su, Yuan, & Zhang, 2013; Wojcikowska et al., 2013), but its significance for LEC‐induced SE is not known. *YUC* overexpression in seedlings is not sufficient to trigger spontaneous SE in seedlings, but rather induces epinastic cotyledon growth (Cheng, Dai, & Zhao, 2006; Kim et al., 2007; Woodward et al., 2005), a typical auxin overproduction phenotype. This suggests that the competence of the underlying tissue is an important determinant for SE induction. LEC2 also upregulates expression of the MADS‐box TF gene *AGL15* (Braybrook et al., 2006) and vice versa (Zheng, Ren, Wang, Stromberg, & Perry, 2009). *AGL15* overexpression facilitates somatic embryo formation from immature zygotic embryos, but does not induce spontaneous SE in seedlings, suggesting that it enhances SE from tissues that are inherently embryogenic (Harding, Tang, Nichols, Fernandez, & Perry, 2003). Notably, both LEC2 and AGL15 activate *INDOLE‐3‐ACETIC ACID INDUCIBLE 30* (*IAA30*), which encodes a non‐canonical Aux/IAA protein (Braybrook et al., 2006; Zheng et al., 2009). The roles of *AGL15* and *IAA30* in LEC2‐induced SE were not determined, but AGL15‐enhanced SE is compromised in the *iaa30* mutant (Zheng et al., 2009).

The relation between *BBM*/*AIL*‐ and *LAFL/AGL15*‐induced SE pathways was unclear for a long time, even though the overexpression phenotypes of these genes are very similar. We recently identified direct BBM targets by chromatin immunoprecipitation in both 2,4‐D‐ and BBM‐induced somatic embryos and showed that BBM binds the promoter regions of *LAFL* and *AGL15* genes (Horstman et al., 2017). BBM‐regulated *LAFL/AGL15* expression is a crucial downstream component of the BBM pathway, as BBM‐induced SE is abolished in the *lec1* and *fus3* mutants and reduced in the *lec2* and *agl15* mutants (Horstman et al., 2017). *BBM* expression is in turn regulated by LAFL proteins: *BBM* expression is reduced in *lafl* mutant seeds, suggesting that LAFL TFs positively regulate *BBM* expression, although it is not clear whether this regulation is direct or indirect (Horstman et al., 2017).

Genetic analysis has shown that AIL proteins interact with auxin pathways throughout plant development (reviewed in Horstman et al., 2014). AIL5 also directly regulates *YUC4* expression to control shoot phyllotaxis (Pinon, Prasad, Grigg, Sanchez‐Perez, & Scheres, 2013). BBM binds to auxin biosynthesis genes (*TAA1*, *YUC3*, and *YUC8*) in somatic embryo tissue, although direct gene regulation was not studied (Horstman et al., 2017).

**Figure 2:**
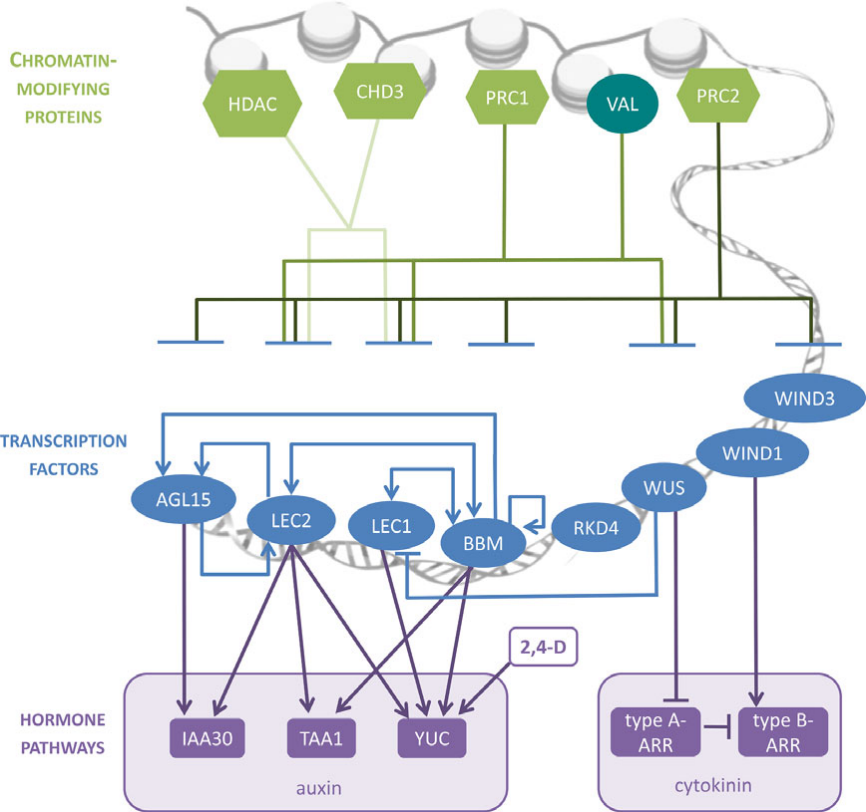
Schematic overview of the molecular regulation of arabidopsis somatic embryogenesis. Chromatin‐modifying proteins (green hexagons) repress or restrict expression of transcription factors (TFs) (blue ovals) during arabidopsis development. The TF VAL, which can recruit PRC1 and HDACs, is indicated on the chromatin level (blue‐green oval). These TFs regulate each other's expression, as well as expression of common target genes involved in the auxin and cytokinin pathways (purple boxes). Evidence for these interactions was not always obtained in somatic embryo tissue, and only a few of the TF−gene interactions have been shown to be direct (see main text for more details). Note that the importance of the TF−hormone interactions for TF‐induced SE was only shown for AGL15‐*IAA30*

These findings indicate that LAFL‐ and AIL‐induced SE are part of an intersecting signaling pathway that converges at the level of auxin (Fig. 2). However, as noted above, enhanced endogenous auxin biosynthesis is not sufficient for spontaneous somatic embryo formation from seedlings, implying that additional components of these pathways are required to induce SE.

The WUS and WIND1 proteins appear to induce SE via a different hormone pathway than AIL/LAFL proteins. WUS and WIND1 pathways converge at the level of cytokinin response rather than auxin biosynthesis and signaling; WUS controls shoot meristem growth by repressing type A *ARR* genes, which are negative regulators of the cytokinin response (Leibfried et al., 2005), while WIND1 stimulates callus formation via type B *ARR* genes, which are positive regulators of the cytokinin response (Iwase et al., 2011) (Fig. 2). The cytokinin response is an important factor for 2,4‐D‐induced secondary SE, as suppression of the cytokinin response through overexpression of type A *ARR* genes led to the formation of fewer and rootless somatic embryos (Su, Liu, Bai, & Zhang, 2015). WUS and WIND1 also interact with the LEC pathway. Sequential activation of WIND1 and LEC2 induces more embryogenic callus in explants than activation of WIND1 or LEC2 alone (Iwase et al., 2015). This suggests that WIND1 overexpression increases the number of competent cells within the explant, which then form somatic embryos in response to LEC2 (Iwase et al., 2015). By contrast, chemical induction of *WUS* reduced *LEC1* expression in *WUS*‐induced somatic embryo tissue (Zuo et al., 2002), suggesting that WUS represses the LEC pathway.

### 4.4 The roles of SE‐inducing transcription factors in 2,4‐D‐mediated SE

Overexpression of the TFs described above can trigger somatic embryo formation in the absence of exogenous hormones, but do they also have a role during 2,4‐D‐induced SE? This point has been addressed in arabidopsis using the immature zygotic embryo and secondary SE systems, using gain‐ and loss‐of‐function mutants.

Single and higher order *lec1*, *lec2*, and *fus3* loss‐of‐function mutants display only mild defects during early zygotic embryogenesis, yet *lec1*, *lec2*, and *fus3* single mutants are severely compromised in 2,4‐D‐induced SE from immature zygotic embryos, in terms of both the number of responding explants and the number of embryos formed per explant. The single mutants produce mainly watery callus and root hairs (Gaj, Zhang, Harada, & Lemaux, 2005), while SE is completely suppressed in *lec*/*fus3* double or triple mutant combinations (Gaj et al., 2005, 2006). Adventitious shoot formation was not affected in these mutants (Gaj et al., 2005, 2006). The discrepancy between the mild defects during early zygotic embryogenesis in *lec1*, *lec2*, and *fus3* mutants and the severe defects observed during somatic embryo induction from immature zygotic embryos suggests that their function during early embryogenesis does not play a major role in SE induction. Rather, SE induction might rely on LEC1/2 and FUS3 function during late embryogenesis, where they are required to maintain embryonic fate. Embryo identity is compromised in *lec* or *fus3* immature zygotic embryos, reducing their competence for 2,4‐D‐induced SE.

The role of *BBM* in 2,4‐D‐induced SE has not been studied, but the role of *PLT2*, a related SE‐inducing *AIL* gene, has been investigated in the arabidopsis 2,4‐D‐induced secondary SE system from embryogenic callus (Su et al., 2015). PLT2 has a role in root meristem maintenance, and *plt2* mutant seedlings have shorter roots than wild‐type seedlings (Aida et al., 2004; Galinha et al., 2007). *PLT2* functions redundantly with *BBM* during zygotic embryogenesis: *plt2* or *bbm* mutant embryos do not show abnormal phenotypes, but the *plt2*/*bbm* double mutant arrests at the two‐celled stage (Galinha et al., 2007; Horstman et al., 2015). Despite the lack of zygotic embryo phenotypes in the *plt2* single mutant, secondary SE from *plt2* immature zygotic embryo explants generates primary and secondary somatic embryos without roots, cotyledons, or shoot apical meristems (Su et al., 2015). Moreover, *plt2* calli also produced fewer secondary somatic embryos, suggesting that *PLT2* is required not only for specification of the root pole in somatic embryos but also for the induction of SE (Su et al., 2015). Single *plt2* mutants do not have an aberrant embryo phenotype, suggesting that the role of *PLT2* in maintaining the root meristem, rather than its (redundant) function during early embryogenesis, is important for SE induction from callus. *PLT2* may be required for the establishment of a lateral root identity in the callus during indirect SE. Whether *PLT2* or other *AIL* genes are also required for 2,4‐D‐induced direct SE is not known.

The requirement of *WUS* for 2,4‐D‐induced SE was investigated using a 2,4‐D system from immature zygotic embryos where embryogenic clusters develop mostly from the shoot apex and to a lesser extent from the cotyledon petioles (Mordhorst, Hartog, El Tamer, Laux, & de Vries, 2002). During seedling development, loss‐of‐function *wus* mutants develop defective shoot apical meristems that are repetitively initiated but prematurely terminated (Laux et al., 1996). Homozygous *wus* explants do not form somatic embryos from the shoot meristem, but do produce embryos from the cotyledons (Mordhorst et al., 2002). Like their zygotic counterparts *wus* somatic embryos subsequently fail to maintain their shoot apical meristem. These data suggest that a functional meristem is required to initiate SE from the shoot apex, but that *WUS* expression is not required for SE induction from cotyledons. The same effect was shown for two other shoot meristem mutants in this system (Mordhorst et al., 2002). In contrast, silencing of *WUS* in a 2,4‐D‐induced secondary embryogenesis system severely reduces the capacity of callus to produce somatic embryos (Su et al., 2009). The different results in these two studies might be explained by the use of different SE culture protocols (direct and indirect), suggesting that WUS is required for indirect SE but not for direct SE.

From these studies we can conclude that 2,4‐D‐induced SE relies on the LAFL and PLT2 TFs, but whether mutations in these genes simply affect the identity of the explant (cotyledon identity in immature zygotic embryos by LAFLs, root or shoot meristem identity in callus by *PLT2* or *WUS*) or whether they are required to initiate processes downstream of 2,4‐D is not clear yet.

## 5 THE ROLE OF CHROMATIN MODIFICATIONS IN SOMATIC EMBRYOGENESIS

### 5.1 Chromatin modifications guide developmental reprogramming

In addition to ectopic expression of the above‐described TFs, loss‐of‐function of certain chromatin remodelers can also induce SE. Chromatin‐modifying factors regulate epigenome reprogramming to change the chromatin states of genes, and are important in particular to guide global transcriptome shifts during phase transitions. In the plant's life cycle, epigenome reprogramming is required for all developmental transitions: from seed to seedling (Molitor, Bu, Yu, & Shen, 2014; Zanten et al., 2014); from vegetative growth to flowering (Hepworth & Dean, 2015); from somatic to reproductive cell during sporogenesis and gametogenesis (Borg & Berger, 2015; Houben, Kumke, Nagaki, & Hause, 2011; She et al., 2013); and after fertilization from gamete to zygotic embryo (Ingouff et al., 2010; Jullien, Susaki, Yelagandula, Higashiyama, & Berger, 2012).

Chromatin modifications affect chromatin compaction, thereby making the chromatin more or less accessible for the transcription machinery. There are three different mechanisms that can alter chromatin accessibility. The first is nucleosome remodeling, a process that is driven by ATP‐dependent nucleosome remodelers such as SWI/SNF‐type proteins. Second, the tails of the histone proteins that constitute the nucleosomes can be subjected to post‐translational modifications, such as acetylation, methylation, ubiquitination, phosphorylation, and glycosylation. Histone marks have either a repressive or an activating effect on gene expression, and usually colocalize to define specific chromatin states that determine gene activity (Roudier et al., 2011; Sequeira‐Mendes et al., 2014). Active genes are usually marked with H3K4me3, H2Bub, H3K9ac, and H3K36me3, while repressed genes are marked with H3K27me3 and H2Aub. The third epigenetic mechanism that influences chromatin accessibility and gene expression is DNA methylation. In plants, the DNA base cytosine can be methylated in different sequence contexts: CG, CHG, and CHH (where H= A, T, or C), each regulated by different DNA methyltransferases. In addition to these three mechanisms, the incorporation of certain histone variants, such as H3.3 or H2A.Z, can also influence gene activity (Stroud et al., 2012).

The chromatin modifications that accompany phase transitions have been well studied in arabidopsis. In plants, the germ cells are initiated from somatic cells in the anther or ovary, which marks the somatic‐to‐reproductive transition. This transition is accompanied by chromatin decondensation, depletion of linker histones, changes in histone variants, and especially in histone modification patterns (She & Baroux, 2015; She et al., 2013). Following fertilization, the chromatin landscape is again drastically modified, mainly by the replacement of histone variants (Borg & Berger, 2015; Ingouff et al., 2010). The subsequent transition from seed to seedling development requires specific chromatin modifications to repress embryo gene expression, and mainly involves histone methylation and acetylation (Bouyer et al., 2011; Molitor et al., 2014; Zanten et al., 2014). The seed‐to‐seedling transition is particularly relevant for understanding in vitro embryogenesis, as loss‐of‐function mutants in chromatin modification proteins that control this phase transition undergo spontaneous SE. Further analyses of these mutants revealed de‐regulation of subsets of the above‐described SE‐inducing genes, placing the involved chromatin remodelers upstream of the SE‐inducing TFs (Fig. 2). Here, we focus on the seed‐to‐seedling transition and discuss the importance of histone H3K27 trimethylation and histone deacetylation for the transcriptional repression of embryo genes.

### 5.2 Histone methylation

The importance of histone methyltransferases in cell fate determination was first shown in animals, where members of the Polycomb repressive complex 2 (PRC2), which deposit K27me3 marks on histone H3, were found to be required for stem cell pluripotency (reviewed in Margueron & Reinberg, 2011). In arabidopsis, double loss‐of‐function mutants in the PRC2 genes *CURLY LEAF* (*CLF*) and *SWINGER* (*SWN*) or *VERNALIZATION 2* (*VRN2*) and *EMBRYONIC FLOWER 2* (*EMF2*) form callus on the shoot apex, which eventually gives rise to indirect somatic embryo formation and ectopic roots (Chanvivattana et al., 2004). Later it was shown that *PRC2* genes are required to repress embryo genes at germination and that mutants in *PRC2* genes fail to terminate the embryonic phase (Bouyer et al., 2011), indicating a role for PRC2 in promoting seedling differentiation. CLF also represses a large number of genes in mature‐green embryos, among which are *AGL15*, *FUS3*, *ABI3*, *AIL5*, and *AIL6/PLT3* (Liu et al., 2016). Recently, Ikeuchi et al. (2015) showed that PRC2 mutant root hairs fail to maintain their differentiated state and form unorganized cell masses and eventually somatic embryos from callus. This is in part due to de‐repression of the PRC2 targets *WIND3* and *LEC2*, as overexpression of both genes induces dedifferentiation of root hair cells (Ikeuchi et al., 2015). These data indicate that PRC2 represses gene expression to promote differentiation and prevent unregulated growth.

The effect of PRC2 on the competence to form somatic embryos depends on the type of explant. In the presence of 2,4‐D, somatic embryos can be efficiently formed from wild‐type immature zygotic embryos, where PRC2 is hardly active, but not from seedling shoots or roots, where PRC2 is expressed and where it represses embryo gene expression (Liu et al., 2016; Mozgová, Muñoz‐Viana, & Hennig, 2017). However, in *clf swn* double mutants, efficient somatic embryo formation can be induced in the shoot apex after 2,4‐D treatment, and even more efficiently after a combined 2,4‐D/wounding treatment. In contrast to the somatic embryo‐like structures that form in the *clf swn* mutant in the absence of treatment (as described above), the somatic embryos formed after 2,4‐D treatment contain root apical meristems and are viable. Transient activation of CLF in a *clf swn CLF‐GR* line abolishes somatic embryo initiation, but does not affect somatic embryo development at a later stage, showing that PRC2 represses the formation and not the outgrowth of somatic embryos from seedling tissues (Mozgová et al., 2017). Interestingly, *clf swn* mutant roots only form somatic embryos when the seedlings are treated with ABA in addition to 2,4‐D, suggesting that the ABA pathway is constitutively active in shoots, but not in roots of PRC2‐depleted mutants (Mozgová et al., 2017).

A tissue‐dependent effect of PRC2 has also been observed during hormone‐induced organogenesis. Organogenesis from leaf explants involves several H3K27me3‐dependent events. First, loss of H3K27me3 marks results in early upregulation of auxin pathway genes, followed by repression of leaf identity gene expression via increased H3K27me3, and finally by root meristem gene expression (He, Chen, Huang, & Xu, 2012). PRC2 function is not required for callus formation from roots; therefore PRC2 seems to be required to eliminate leaf identity by silencing leaf‐specific genes. Thus PRC2 can have a negative or positive effect on regeneration, depending on whether the identity of the explant needs to be retained (embryo identity after germination for SE) or silenced (leaf identity during callus formation for organogenesis) to allow regeneration. These examples strengthen the idea that the outcome of downregulation of somatic embryo‐repressing chromatin remodelers or overexpression of SE‐inducing genes strongly depends on the tissue context in which it takes place.

A second Polycomb repressive complex, PRC1, functions together with PRC2 to repress embryo gene expression during germination. PRC1 is found in plants and animals, where it represses gene activity by histone H2A ubiquitination (H2Aub). Double mutants in the PRC1 subunits AtBMI1a/b and AtRING1a/b show retarded germination, fail to form true leaves, and develop callus‐ and embryo‐like structures (Bratzel, Lopez‐Torrejon, Koch, Del Pozo, & Calonje, 2010; Chen, Molitor, Liu, & Shen, 2010). This phenotype is associated with upregulation of *LAFL* gene expression, as well as ectopic expression of the stem cell regulators *WUSCHEL RELATED HOMEOBOX 5* (*WOX5)*, *BBM*, *SHOOT MERISTEMLESS* (*STM*) and *WUS* (Chen et al., 2010; Yang et al., 2013). The same set of genes is de‐repressed in *val1/2* (*vp1/abi3‐like 1/2*) double mutants, which also fail to form true leaves and develop embryo‐like proliferations in the root and shoot apical meristem regions (Suzuki, Wang, & McCarty, 2007; Yang et al., 2013). VAL1 and VAL2 are B3 domain TFs, as are the SE inducers ABI3, FUS3, and LEC2, but the VAL proteins also possess CW and PHD domains that can associate with chromatin factors (Suzuki et al., 2007). VAL proteins are required for PRC1‐mediated deposition of H2Aub at seed maturation genes and *BBM* (Yang et al., 2013). PRC1 is probably recruited to its target genes by VAL proteins, since the VAL‐binding motif is enriched in promoters of PRC1 target genes (Merini et al., 2017) After the initial silencing by PRC1/VAL, repression is maintained by PRC2‐mediated H3K27me3 deposition. Thus, VAL repression of *LAFL* genes is mediated by recruitment of Polycomb complexes. To emphasize the special role of VAL, we included the factor at the same level as the chromatin remodelers in Figure 2.

### 5.3 Histone deacetylation

Histone H3 and H4 acetylation is associated with a positive effect on gene transcription. The level and position of histone acetylation is tightly regulated by the activity of histone acetyl transferases (HATs) and histone deacetylases (HDACs). The first indication that histone deacetylation plays a major role during SE was provided by Tanaka, Kikuchi, and Kamada (2008), who showed that the HDAC inhibitor trichostatin A (TSA) induces growth arrest and upregulation of the embryonic markers *LEC1*, *FUS3*, and *ABI3* in germinating arabidopsis seeds, suggesting that the embryo failed to make the transition to seedling growth. This effect could be phenocopied by the *hda6*/*hda19* histone deacetylase double‐repression line, which also formed somatic embryos on the leaves (Tanaka et al., 2008). Two reports unravel the mechanism by which HDA6 and HDA19 repress embryo gene expression. HDA19 specifically interacts with VAL2 (Zhou et al., 2013), while HDA6 interacts with VAL1 (Chhun et al., 2016), and both VAL1 and VAL2 interact with the repressive CDK8 module of the Mediator complex. VAL1 and VAL2 can therefore recruit both histone deacetylases and the repressive form of the Mediator complex to the *LAFL* genes to repress their expression (Chhun et al., 2016). Strikingly, VAL proteins appear to recruit both Polycomb group proteins and HDACs to achieve repression of the *LAFL* genes (see Fig. 2).

The CHD3‐type ATP‐dependent SWI/SNF chromatin‐remodeling factor PICKLE (PKL) also mediates cross‐talk between histone methylation and acetylation. The *pkl* mutant seedlings retain embryonic traits after germination and produce somatic embryos from a number of seedling tissues, coinciding with de‐repression of the *LEC* genes (Henderson et al., 2004; Ogas, Kaufmann, Henderson, & Somerville, 1999). CHD3 proteins are associated with histone deacetylases in animals (Torchy, Hamiche, & Klaholz, 2015), but loss of *PKL* in plants was found to affect global H3K27me3 levels, also at the *LEC1* and *LEC2* loci, rather than acetylation levels (Zhang et al., 2008). However, HDAC activity has also been shown to be required for certain *pkl* phenotypes in plants (Fukaki, Taniguchi, & Tasaka, 2006), and TSA treatment could to a certain extent mimic *pkl* phenotypes in arabidopsis calli (Furuta et al., 2011), suggesting that PKL may function by guiding both H3K27me3 and histone deacetylation. Thus, it appears that the combination of PRC2‐ and HDAC‐induced repression ensures the silencing of the embryonic program, but loss of either PRC2 or HDAC activity can be sufficient to induce SE. It would be interesting to determine whether the combination of both TSA treatment and loss of PRC2 can further induce SE potential.

A role for histone acetylation in promoting embryo identity has also been described in in vitro embryogenesis systems in other plants. Germinating spruce somatic embryos treated with TSA maintain their embryo identity rather than converting to seedlings (Uddenberg et al., 2011). TSA was also found to enhance haploid embryo induction from heat‐stressed in vitro cultured male gametophytes in *B. napus* (Li et al., 2014). These data indicate that histone deacetylation is a conserved mechanism in plants to repress embryo identity outside the seed. Similar to H3K27me3, levels of histone acetylation and the activity of HDACs has also been found to change after hormone‐induced indirect SE (De‐la‐Peña, Nic‐Can, Galaz‐Ávalos, Avilez‐Montalvo, & Loyola‐Vargas, 2015; Lee, Park, Jung, & Seo, 2016), providing indirect evidence that histone deacetylation may also play a role in the reprogramming of cells in the early stages of indirect SE.

### 5.4 Role of chromatin modifications in different regeneration systems

Histone methylation (H3K27me3) and histone acetylation play an important role in SE by regulating the expression of embryonic and meristematic genes. The extent to which chromatin reprogramming is required prior to SE probably depends on the explant and culture system in which SE is induced, and might also require additional chromatin modifications such as changes in DNA methylation, which are often associated with SE (De‐la‐Peña et al., 2015). Direct SE appears to involve less reprogramming than indirect SE and probably occurs in cells where totipotency genes are transcriptionally accessible. The involvement of more extensive chromatin remodeling during indirect SE is suggested by the differential expression of many chromatin modifiers after 2,4‐D‐induced callus formation from protoplasts, leaves, hypocotyls, and roots (Chupeau et al., 2013; Xu et al., 2012).

## 6 CONCLUSIONS AND FUTURE PERSPECTIVES

Plant cell totipotency has intrigued scientists for decades. The first protocols used stress or growth factor treatments for SE induction and the process was studied at the histological level. SE can now be induced by embryo‐ and meristem‐expressed TFs, as well as TFs involved in wound repair. Molecular analysis identified cross‐regulation between the different SE‐inducing TFs, as well as a role for hormone pathways in SE induction. Genetic analysis has shown that mutations in SE‐inducing TFs reduce the efficiency of 2,4‐D‐induced SE, but it is not clear whether the TFs are required downstream of 2,4‐D or whether SE is impaired due to altered explant identity. An inducible gene silencing approach could be used to address this point.

Somatic embryos can develop directly or indirectly via a callus phase. Whether this callus has lateral root identity, similar to organogenic callus, is a topic for future studies. Our work on BBM suggests that the transcriptional accessibility of embryo genes in different tissues might determine whether embryos are formed via direct or indirect SE. Both types of SE, as well as organogenesis, coexist in different 2,4‐D‐ and TF‐induced tissue culture systems, suggesting that these types of regeneration are mechanistically linked. A study on BBM has shed a light on this phenomenon, as BBM induces organogenesis or SE depending on its dose, but whether the same or a different set of genes is involved in these different types of regeneration is not known. Finally, multiple studies show that SE‐inducing genes are repressed through histone methylation and deacetylation during the transition from embryo to seedling development. Loss‐of‐function mutants in the chromatin modification proteins that regulate these marks undergo spontaneous SE, which is accompanied by upregulation of key embryo identity genes, including genes for SE‐inducing TFs. Whether there are major differences between the chromatin‐level reprogramming that occurs during direct and indirect SE and between SE and adventitious organogenesis remains to be investigated. The fact that only a small fraction of the cells in an explant are induced to form somatic embryos and that these embryogenic cells are difficult to recognize at an early stage complicates a detailed study of the chromatin changes, such as the one performed by She et al. (2013) on cells that undergo the somatic‐to‐reproductive fate transition. It would be interesting to investigate the cell‐specific dynamics of chromatin modifications after the induction of SE by 2,4‐D, stress, or ectopic expression of different TFs using techniques such as fluorescence‐activated cell sorting (Carter, Bonyadi, & Gifford, 2013), where cells marked by an embryo reporter can be specifically selected.

## CONFLICT OF INTEREST

The authors declare no conflict of interest.
